# Supplementary material for: Stance Phase Gait Training Post Stroke Using Simultaneous Transcranial Direct Current Stimulation and Motor Learning-Based Virtual Reality-Assisted Therapy: Protocol Development and Initial Testing
Source: Brain Sci. 2022 May 28;12(6):701. doi: 10.3390/brainsci12060701 (PMC9221094; doi:10.3390/brainsci12060701)
Supplement: Supplementary file 1 [file brainsci-12-00701-s001.zip › brainsci-1733432-supplementary.pdf]

Table S1: Changes in each clinical measure score from baseline to post-treatment for each user.

User	Fugl-Meyer (Points)		Gait Speed (m/s)		TUG (s)		FGA (Points)	
	Pre	Post	Pre	Post	Pre	Post	Pre	Post
S1 ●	28	30	1.03	1.18	11.50	8.79	17	23
S2 ◆	22	23	0.96	1.23	13.61	11.94	14	18
S3 ▲	17	22	1.16	1.61	7.73	7.68	8	14
S4 ■	17	25	0.39	0.47	27.24	23.75	11	14
S5 ★	22	27	1.11	1.44	11.92	8.15	16	20
Mean (SD)	21.2 (4.5)	25.40 (3.21)	0.93 (0.31)	1.19 (0.44)	14.40 (7.49)	12.06 (6.74)	13.2 (3.7)	17.80 (3.90)
